# A Case of Primary Lung Adenocarcinoma With Two Uncommon Presentations: Neurological Paraneoplastic Syndrome and Pericardial Effusion

**DOI:** 10.7759/cureus.47753

**Published:** 2023-10-26

**Authors:** Diana V Castro, Jorge Mendes, Luísa Pinto, Nuno Ferreira, Luís Pereira

**Affiliations:** 1 Intensive Care Unit, Centro Hospitalar de Leiria, Leiria, PRT

**Keywords:** non-small cell lung cancer, intensive care medicine, pericardiocentesis, pericardial effusion, paraneoplastic neurologic syndrome, lung cancer

## Abstract

Lung cancer is the second most common cancer worldwide and remains the first cause of cancer death. The diagnosis of lung cancer is mostly made following evaluation for respiratory signs and symptoms but sometimes the first presentation may be atypical. Some symptoms may be related to the invasion of adjacent structures and others caused by an autoimmune-mediated process when cross-reactivity between tumor antigens and normal nervous tissues is responsible for paraneoplastic syndromes. We present a case of a young woman with a smoking history who first manifested with two uncommon presentations of lung cancer: a paraneoplastic neurological syndrome and a hemorrhagic pericardial effusion with cardiac tamponade.

## Introduction

Lung cancer has a high prevalence and remains the first cause of death of oncologic origin and this is mainly related to smoking habits [1,2]. Due to the great number of cases, there are many atypical manifestations described. The diagnosis of lung cancer is mostly made following evaluation for respiratory signs and symptoms [3]. Clinical manifestations can be related to the location of the primary tumor, the invasion of adjacent structures, metastatic disease, and paraneoplastic manifestations [1]. Individuals with primary lung cancer are usually divided into two groups: small-cell lung cancer and non-small-cell lung cancer. The latter corresponds to more than 80% of cases [1-3]. Pericardial effusion with cardiac tamponade is a rare presenting sign and a life-threatening manifestation of advanced malignancy. In 4-7% of cases, acute pericardial disease is the first sign of an occult malignancy, usually a primary lung cancer [3-5]. On the other hand, paraneoplastic neurological syndromes (PNS) can commonly occur due to lung cancer and most of them are present in small-cell lung cancer patients. In fact, in 50-85% of cases, PNS develops before any other symptom of cancer [1,6]. We present a case of a young woman with a smoking history who first presented with these two rare and atypical signs, a PNS and a pericardial effusion, before any common symptoms of lung cancer.

## Case presentation

A previously healthy 45-year-old woman presented to the emergency room with a two-day history of severe frontal headache (rated an 8/10). This was associated with paresthesias of the right hemiface, ptosis on the right eye, and hypoesthesia of both upper limbs. She denied fever, alteration of consciousness, fall, or trauma. She had a weight loss of 6 kg in one month. She denied cough, chest pain, abdominal pain, hematochezia, hematemesis, constipation, or changes in the urinary tract. She also denied any recent travel or any other kind of exposure or drug consumption. She was a smoker of 20 packs/year. The patient’s blood pressure at the time of admission was 144/64mmHg, heart rate was 62 beats per minute in sinus rhythm, respiratory rate was 16/minute, and blood oxygen saturation was 97% on room air. Her tympanic temperature was 36.1ºC. The neurological examination revealed a complete ophthalmoparesis of right oculomotor, binocular diplopia, without facial asymmetry, gag reflex diminished bilaterally, and distal monoparesis of the left upper limb (Grade 4 - active movement against gravity and resistance). The remaining neurological examination was unremarkable. Cardiac and pulmonary auscultation and abdominal palpation showed no alterations.

Laboratory studies revealed a leukocytosis with neutrophilia. Liver enzymes, electrolytes, and kidney function were unremarkable. Hepatitis B virus, hepatitis C virus, and human immunodeficiency virus (HIV) serological tests were negative. Autoimmune workup including antinuclear antibody (ANA), anti-dsDNA antibody, and Sjögren A and B antibodies were negative. Serum electrophoresis was without suspected monoclonal component.

Cranial and cervical contrast-enhance computed tomography (CT) and magnetic resonance imaging didn’t show any lesion or any acute alteration. Lumbar puncture showed an albumin-cytological dissociation (with 1794 mg/L proteins and 45/mm^3^ leukocytes); microbiology, infectious and autoimmune serologies, antineuronal antibodies, cells neoplastic, antiganglioside antibodies had no significant alterations. An electroneuromyography was performed, which demonstrated reduced motor nerve conduction amplitude of the right facial nerve, and loss of motor units with a mixed/poor pattern with a tendency to simplification in the trapezius, orbicularis oris, and tongue muscles, without signs of active denervation. The rest of the electroneuromyographic evaluation was normal. Repetitive stimulation study was normal. These findings of motor axonal neuropathy, associated with the clinical issues, may be suggestive of the atypical Miller-Fisher-Guillain Barré variant with onset in the brainstem.

On the second day of hospitalization, in addition to the neurological deficits already described, the patient developed dysarthria, dysphagia, and dysphonia. Acute inflammatory neuropathy was assumed and immunoglobulin therapy was initiated with no improvement in neurological examination. On day six of hospitalization, the patient developed dyspnoea with a need for oxygen therapy (fraction of inspired oxygen (FiO2) 60%) and increased work of breathing with tachycardia, a normal blood pressure but a lactate of 2.4 mmol/L and jugular venous distention.

**Figure 1:**
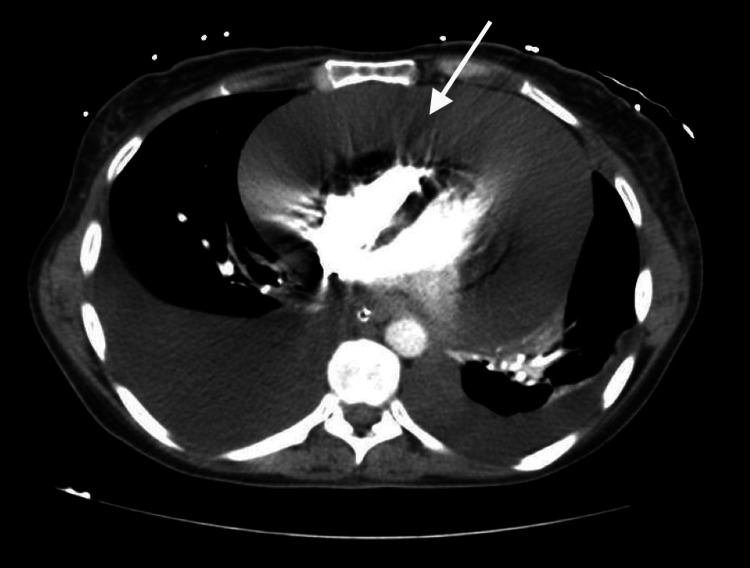
Pleural and pericardial effusion (arrow) on CT thorax

Chest X-ray revealed a prominent cardiac silhouette. CT of the thorax revealed a pericardial effusion of 20-50 mm thickness and bilateral pleural effusion. There were no pulmonary nodules (Figure 1).

The patient was immediately admitted to the intensive care unit (ICU). Echocardiography was obtained which showed normal left and right ventricular systolic functions, normal valvular structures, a large circumferential pericardial effusion with right ventricular compression, and right atrial inversion which is a sign of cardiac tamponade (Video 1).

**Video 1:** Pericardial effusion with signs of cardiac tamponade.

**Figure 2:**
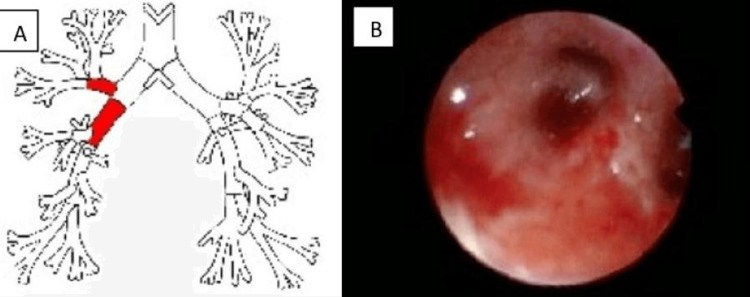
Lesion location (panel A) with an image of right upper lobe with mucosa with infiltrative signs and enlargment of the spur between the right upper lobe bronchus and the intermediate bronchus (panel B).

A pericardiocentesis was performed with the removal of 800 mL of serosanguineous fluid and with a significant improvement in the patient’s symptoms. A sample of pericardial fluid was sent for evaluation. A bronchoscopy was also performed (Figure 2).

After all study results, bronchoscopy biopsies and pericardial fluid evaluation showed a non-small-cell carcinoma, favor adenocarcinoma. The immunohistochemical study revealed positivity for cytokeratin (CK) 7, thyroid transcription factor 1 (TTF-1), and Napsin-A and negativity for p40 and CK 20, favoring the diagnosis of primitive lung adenocarcinoma. A mutation was found in the *KRAS/NRAS* gene; this was associated with a worse response to treatment with epidermal growth factor receptor (EGFR) inhibitors. The expression of PD-L1 22C3 was less than 1%, which meant a low response to immune checkpoint inhibitor therapy (pembrolizumab). Cranial and thoracic-abdominopelvic CT did not demonstrate metastatic disease. The patient started a cycle of chemotherapy with carboplatin and pemetrexed and there was no pleural effusion recurrence, but there were no significant improvements in PNS, at the time of discharge from the ICU.

## Discussion

Lung cancer stands as the second most prevalent cancer globally and remains the leading cause of cancer-related fatalities, with an estimated 1.8 million deaths in 2018 [1,2]. Tobacco is the greatest risk factor and it is responsible for the majority of lung cancer cases [2,7,8]. Most patients present for diagnostic evaluation because of respiratory signs or symptoms or an incidental finding on chest imaging like a nodule or a mass. The most common symptoms are chronic cough, hemoptysis, dyspnoea, and chest pain [1-3,7,8].

In non-neurological neoplasms, neurological manifestations may arise from factors like chemotherapy, malnutrition, infection, direct tumor invasion of the nervous system, or autoimmune-mediated processes known as PNS [6,9]. PNS form a group of rare neurological diseases caused by the immune effect of the primary tumor in the neurological system of the patient [10]. This autoimmune-mediated process involves the expression of neuronal antigens by the primary tumor, resulting in the inappropriate recognition of normal nervous system elements as foreign [6,9,10]. This cross-reactivity between tumor antigens and the normal nervous system is responsible for various PNS (for example sensory neuronopathy, opsoclonus-myoclonus, Lambert-Eaton myasthenic syndrome) [1,10,11]. The presence of onconeural antibodies is important for PNS diagnosis (examples anti-Hu, Yo, CV2, Ri, Ma2, or amphiphysin) [12]. However, PNS may occur without onconeural antibodies, or all hospitals may not be able to test them. Their presence isn't a necessary condition for PNS diagnosis [12]. An acute sensorimotor neuropathy, as observed in this case, falls under the category of non-classical PNS according to diagnostic criteria recommended by Graus et al. According to these authors, despite being a non-classical syndrome without onconeural antibodies, the presence of cancer makes PNS possible [12]. PNS is relatively rare, affecting less than 1% of all cancer patients, but lung cancer is the most common cancer associated with PNS, with approximately 50-80% of patients experiencing neurological manifestations [10].

Pericardial disease is detected in up to 20% of cancer in autopsy studies and in 4-7% of cases, it is a first sign of an undiscovered malignancy, usually a primary lung cancer. This suspicion is greater if there is pleural effusion [3-5]. As described in our case report, pleural and pericardial effusions constitute one of the presenting symptoms in addition to PNS. Pericardial effusion might develop as a result of direct or metastatic spread of the primary tumor or an adverse effect of some therapies or radiation. In the patient in the current report, a young woman without any recent cardiac surgery and with a hemorrhagic pericardial effusion, there are several differential diagnoses that need to be considered: metastatic cancer, lymphoma, autoimmune disorders such as systemic lupus erythematosus and infectious diseases such tuberculosis and HIV. All of these alternative diagnoses were ruled out, with lung cancer emerging as the most likely cause. We must note that hemorrhagic pericardial effusion accompanied by tamponade is a sign of an undiagnosed and advanced malignancy with a poor prognosis [3-5,13,14].

The patient had a diagnosis of non-small-cell lung cancer with two uncommon presentations: a PNS and a pericardial effusion accompanied by tamponade. In PNS, immunomodulatory treatment may be considered, even when the underlying malignancy cannot be identified. Possible approaches include intravenous immunoglobulin, plasma exchange, corticosteroids, or rituximab [12,15]. On the other hand, for a patient with a pericardial effusion with imminent tamponade, timely drainage is essential. The recurrence of pericardial effusion after pericardiocentesis is a common problem and, usually, it is recommended to perform a pericardial window [16]. The most appropriate treatment is to treat the underlying cause.

## Conclusions

PNS, although rare, turns out to be relatively common in lung cancers such as pericardial effusion. Thereby, given the combination of both manifestations, a diagnosis of lung cancer must be strongly considered on the differential diagnosis of this case report. Although chest imaging does not raise the suspicion of lung cancer, we actively search the diagnosis by fibroscopy.

An important aspect of our case, and of similar instances, is that PNS and pericardial effusions can manifest prior to the appearance of primary malignancy signs and symptoms. This clinical scenario offers a valuable opportunity for early diagnosis and treatment of an undiagnosed malignancy, potentially altering the prognosis of the disease.

## References

[1] Milanez FM, Pereira CA, Trindade PH, Milinavicius R, Coletta EN (2008). Lung adenocarcinoma, dermatomyositis, and Lambert-Eaton myasthenic syndrome: a rare combination. J Bras Pneumol.

[2] Remon J, Soria JC, Peters S (2021). Early and locally advanced non-small-cell lung cancer: an update of the ESMO clinical practice guidelines focusing on diagnosis, staging and systemic and local therapy. Ann Oncol.

[3] Dessalegn N, Felux K, Seid E, Mohammed A (2022). Primary lung adenocarcinoma presenting as cardiac tamponade in a 40-year-old non-smoker. Cureus.

[4] Babu RS, Lanjewar A, Jadhav U, Wagh P, Aurangabadkar G, Upadhyay P (2022). A case series of malignant pericardial effusion. J Family Med Prim Care.

[5] Neves MB, Stival MV, Neves YC (2021). Malignant pericardial effusion as a primary manifestation of metastatic colon cancer: a case report. J Med Case Rep.

[6] Koike H, Sobue G (2013). Paraneoplastic neuropathy. Handb Clin Neurol.

[7] Mao Y, Yang D, He J, Krasna MJ (2016). Epidemiology of lung cancer. Surg Oncol Clin N Am.

[8] Nicholson AG, Tsao MS, Beasley MB (2022). The 2021 WHO classification of lung tumors: impact of advances since 2015. J Thorac Oncol.

[9] Giglio P, Gilbert MR (2010). Neurologic complications of cancer and its treatment. Curr Oncol Rep.

[10] Soomro Z, Youssef M, Yust-Katz S, Jalali A, Patel AJ, Mandel J (2020). Paraneoplastic syndromes in small cell lung cancer. J Thorac Dis.

[11] Kesner VG, Oh SJ, Dimachkie MM, Barohn RJ (2018). Lambert-Eaton myasthenic syndrome. Neurol Clin.

[12] Graus F, Delattre JY, Antoine JC (2004). Recommended diagnostic criteria for paraneoplastic neurological syndromes. J Neurol Neurosurg Psychiatry.

[13] Nardi-Agmon I, Zer A, Peysakhovich Y, Margalit I, Kornowski R, Peled N, Iakobishvili Z (2022). Development of pericardial effusion in non-small cell lung cancer is associated with the presence of EGFR/ALK mutations. Isr Med Assoc J.

[14] Mehmood MA, Bapna M, Siddiqa A, Haider A, Saad M (2020). Hemorrhagic pericardial effusion leading to cardiac tamponade: a rare initial presentation of adenocarcinoma of the lung. Cureus.

[15] Pelosof LC, Gerber DE (2010). Paraneoplastic syndromes: an approach to diagnosis and treatment. Mayo Clin Proc.

[16] Li BT, Pearson A, Pavlakis N (2014). Malignant cardiac tamponade from non-small cell lung cancer: case series from the era of molecular targeted therapy. J Clin Med.

